# The therapeutic effect of melatonin on female offspring ovarian reserve and quality in BALB/c mice after exposing their mother to methamphetamine during pregnancy and lactation

**DOI:** 10.22038/IJBMS.2022.66660.14636

**Published:** 2023-02

**Authors:** Negar Osatd-Rahimi, Ehsan Saburi, Sareh Karimi, Arad Boustan, Alireza Ebrahimzadeh-bideskan

**Affiliations:** 1 Department of Anatomy and Cell Biology, School of Medicine, Mashhad University of Medical Sciences, Mashhad, Iran; 2 Medical Genetics and Molecular Medicine Department, School of Medicine, Mashhad University of Medical Sciences, Mashhad, Iran; 3 Medical Genetics Research Center, Mashhad University of Medical Sciences, Mashhad, Iran; 4 Department of Medical Biotechnology and Nanotechnology, Faculty of Medicine, Mashhad University of Medical Sciences, Mashhad, Iran; 5 Applied Biomedical Research Center, School of Medicine, Mashhad University of Medical Sciences, Mashhad, Iran

**Keywords:** Lactation, Melatonin, Methamphetamine, Ovarian reserve, Pregnancy

## Abstract

**Objective(s):**

Nowadays, methamphetamine (METH) abuse as a psychotropic drug is increasing. There is insufficient information about its adverse effects on the ovarian reserve of the next generation. Herein, we tried to investigate the effect of METH abuse during pregnancy and lactation and, subsequently, the therapeutic effect of melatonin on ovarian reserve in offspring.

**Materials and Methods:**

In the present study, BALB/c pregnant female mice were divided into 3 groups: Control, Saline, and METH (5mg/Kg). METH was injected during pregnancy and lactation, and the female offspring of each group was divided into 2 subgroups: A) treated with 10 mg/kg Melatonin daily until puberty (6 weeks old) and B) received distilled water. The animals were sacrificed at 6 weeks of age, and blood samples were collected for hormonal assessments; the right ovaries were removed and fixed for TUNEL and Hematoxylin & Eosin staining, and the left ovaries were removed and stored for gene expression and oxidative stress evaluation.

**Results:**

In the MTEH group, two indicators of ovarian reserve (including anti-Müllerian hormone (AMH) and primordial follicle, and Cyclin D1 (CCND-1) and proliferating cell nuclear antigen (PCNA) genes expression significantly decreased, and the oxidative stress and apoptosis significantly increased in comparison with other groups. After lactation in the MTEH group, melatonin treatment significantly improved the ovarian reserve and gene expression and declined apoptosis and oxidative stress.

**Conclusion:**

METH abuse during pregnancy and lactation decreased ovarian reserve in offspring. The administration of melatonin as an anti-oxidant agent after lactation can counteract the adverse effects of METH on offspring ovaries.

## Introduction

Methamphetamine (METH) is known as a psychoactive and powerful addictive drug that rapidly enters the central nervous system (1). Increasing adolescent METH abuse has become a crucial global health problem (2). METH changes the lift mood, alertness, energy levels and concentration in the short-term and chronic use of METH leads to psychosis, depression, delusions, and violent behavior. Also, METH influences the nervous system (CNS), the immune system, and the gastrointestinal system (3). One of the harmful effects of METH on the CNS is through the hypothalamic-pituitary-gonad axis, which reduces the levels of luteinizing hormone (LH) and then induces disorders in the male reproductive system, such as lowering the sperm count and normal sperm and increasing the apoptotic cells in seminiferous tubules (2, 4). Abuse of METH in females during pregnancy leads to increased maternal and fetal mortality and morbidity and other side effects such as gestational hypertension and preeclampsia, intrauterine fetal death, and pre-term labor (5). Also, because of disruption in the hypothalamic-pituitary-gonadal axis following METH abuse, the menstrual cycle becomes irregular (6). Due to its low molecular weight and high-fat solubility, METH not only crosses the placental barrier easily (within 30 sec of its administration) but also accumulates and is secreted in breast milk during lactation (7). In addition, METH increases the expression of P53 as an apoptotic factor and elevates apoptotic proteins such as BAD, BAX, and BID while decreasing anti-apoptotic proteins, including Bcl2 and Bcl-xl (8). Moreover, METH induces mitochondrial damage and oxidative stress, which impact subfertility in both males and females (9, 10). 

The granulosa cells synthesize the anti-Mullerian hormone (AMH). This glycoprotein hormone is a member of the transforming growth factor-β (TGF-β) superfamily, known as a serum biomarker for assessing ovarian reserve, and demonstrates primordial follicles numbers (11). METH reduces the level of AMH by decreasing the number of follicles (2). Reactive oxygen species (ROS) are produced During the ovarian physiological metabolism. For normal ovarian functions and to prevent injury and then infertility, ROS must be removed and balanced between pro-oxidant and anti-oxidant (12). 

Melatonin (MLT) is a neuro-hormone and anti-oxidant compound secreted by the pineal gland. Melatonin is a strong anti-oxidant that is effective in declining oxidative stress and apoptosis; furthermore, it can decrease the toxicity of METH by its anti-oxidant potential (13, 14). In long-term decline in ovarian aging, melatonin treatment also protects the pool of follicles, oocyte quantity, and quality (15). 

Based on previous studies, abuse of METH has negative effects on women’s reproductive organs but based on our knowledge, the information about the effect of METH exposure during pregnancy and lactation on female offspring reproductive system and its mechanism(s) is deficient. According to the importance of primordial follicles as ovarian reserves that develop during the embryonic period, this study was designed to investigate the adverse effects of METH abuse during pregnancy and lactation on ovarian reserve and quality in the next generation and the therapeutic effect of melatonin subsequently.

## Materials and Methods

***Animals and treatment***

All the animals were housed in the standard conditions (12 hr light and dark cycle, at a temperature of 22-24 °C, 50 % relative humidity, and free access to food and drinking water). All institutional and national guidelines and ethical considerations were followed by the National Institute of Health Guide for the Care and Use of Laboratory Animals and approved by the Ethics Committee at Mashhad University of Medical Sciences, Mashhad, IRAN (IR.MUMSM.MEDICAL.REC 1398.772).

For this study, mature male and female BALB/c mice were obtained from the animal house of Mashhad University of Medical Sciences. For mating, 2 virgin mature female mice and 1 male mouse (1:2) were placed in separate cages. The next morning, the vaginal plaque was checked to confirm successful mating and pregnancy. Finally, 24 pregnant female BALB/c mice were divided into 3 groups:

1- Control group (Ctrl): without any intervention during pregnancy and lactation period (n=8), 2- Saline group (Sal): the animals received daily intraperitoneal (IP) injections of normal saline during pregnancy and lactation period (n=8), 3- METH group (Meth): the animals received daily IP injections of 5 mg/kg METH during pregnancy and lactation (n=8) (16). 

**Figure 1:**
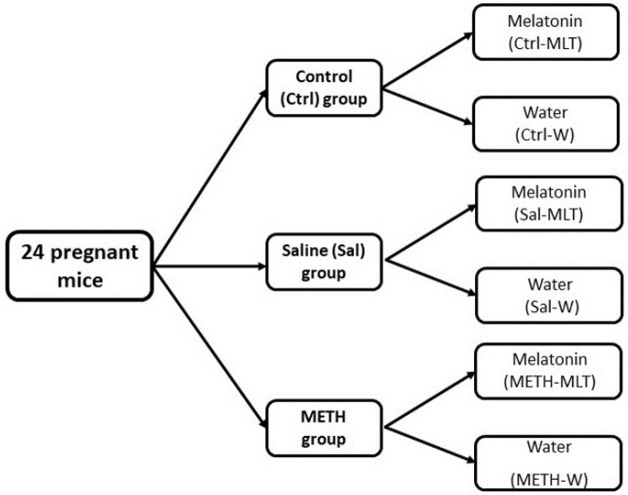
Study design diagram

At the end of pregnancy and lactation, in each group, 3-weeks-old female offspring were separated from their mother and were divided into 2 subgroups: A) the offspring received 10 mg/kg melatonin (MLT) (Nature Made tablet- USA) daily by gavage at the hours of darkness for 3 weeks until puberty (6 weeks old) and B) the offspring received water by gavage (W) (Figure 1). When the female offspring reached puberty (6 weeks of age), they were anesthetized with xylazine and ketamine, and then blood samples were collected from the apex of the cardia for hormonal tests, and the ovaries were removed, right ovaries were fixed in formalin for histological study and the left ovaries were removed and stored in RNA lather and normal saline (-80 °C) for real-time PCR and oxidative stress assessment respectively (17). 

***Hormone assay***

The plasma was separated by centrifugation of blood at 3500 rpm for 15 min. After centrifugation, the plasma was collected and reserved at -80 ℃. The level of estrogen (E2), progesterone (P4), and AMH hormones were assayed by the enzyme-linked immunosorbent assay (ELISA) method (18).

***Biochemical assessment***

To prepare homogeneous tissue, we homogenized 100 mg of tissue with 1 ml of cold phosphate buffer (pH = 7.4) by mechanical homogenizer on the ice bag, then centrifuged for 10 minutes at 10000 rpm, and finally, the supernatant was removed and stored in -80 °C. 

***Malondialdehyde (MDA) level measurement***

In this study, MDA was measured by the TBARS method. MDA, a terminal product of lipid peroxidation, reacts with thiobarbituric acid (TBA) and produces a red complex with an optical density (OD) peak at 532 nm. Its absorption at 535 nm was read using a spectrophotometer. The MDA concentration level was calculated by using the following formula (19):

C(M)=Absorbance/1.56 *10^5

***SOD level measurement***

The activity of SOD was measured according to the Madesh and Balasubramanian method. This method is a colorimetric assay based on superoxide generation by pyrogallol autoxidation and inhibition of tetrazolium salt conversion into red formazan dye by SOD. Thereafter, the colorimetric changes were read at 570 nm using an ELISA device (20).

***Thiol level measurement***

A dithiol nitro benzoic acid (DTNB) reagent was used to measure the thiol groups. This reagent reacts with SH groups of thiol to form the yellow complex of 1, 3, 5-Trinitrobenzene TNB, which has an absorption peak at 412 nm. One ml of the Tris-EDTA buffer was added to the supernatant of homogenized tissue (pH = 8.6), and then sample absorbance was measured at 412 nm in the Tris-EDTA buffer alone (A1). Then 20 μl of DTNB reagent was added to the solution and stored at 15 °C. The sample absorption was then read again (A2). DTNB **absorption** alone was considered as (B), and the total concentration was calculated based on the following formula (19) :

Total thiol concentration (mM)=(A2-A1-B) × 1.07/0.05 × 13.6

***Catalase *** (CAT) ***level measurement***

The activity of the CAT enzyme was measured by Aebi’s method following the decomposition of hydrogen peroxide (H_2_O_2_) at a wavelength of 240 nm. The reaction was started by adding 30 mM H_2_O_2_ to an appropriate volume of tissue homogeneity in 50 mM sodium phosphate buffer (pH=7). The absorbance was read within 3 min at a wavelength of 240 nm, and the specific activity was calculated in units per milligram of protein per min (units/mg protein/min) (21).

***Histological methods***

 The right ovaries were fixed in Normalin 10% (10 cc formalin + 90 cc normal saline) for 48 hr, dehydrated with an ascending ethanol series, cleared with xylene, and then embedded in paraffin. The paraffin blocks were cut into 5 μm thickness with 25 μm intervals using a microtome (Leitz 1512, Germany), and then 5 sections in each animal were selected by systematic randomized method and put on a glass slide. The selected tissue sections were deparaffinized with xylene, rehydrated through descending concentrations of ethanol, and rinsed for 10 min in water to be ready for staining (22).

***Hematoxylin*** ***&*** ***Eosin (H&E) staining and follicle counting***

The sections were stained with H&E**, **evaluated, and photographed using a light microscope (B51, Olympus, Japan) equipped with a high-resolution camera with a ×40 objective lens. The photomicrographs taken were transferred to the computer for further analysis. The morphology of follicles in different growth stages was evaluated (23).

The primary follicles, encapsulated by a single layer of squamous follicular cells, were considered primordial. The follicles surrounded by a single layer of cuboidal follicular cells were identiﬁed as primary follicles. The atretic follicles with their follicular cells tend to degenerate with the basement separated from the granulosa cells. Only the follicles with oocytes were counted to avoid duplicate follicle counting.

***TUNEL assay***

Apoptotic cells were detected by the TUNEL Kit (Roche, Germany). First, the tissue sections were deparaffinized with xylene, rehydrated with descending degree of ethanol, and then washed in 0.1M phosphate-buffered saline (PBS) 3 times for 5 min. The sections were put in 3% H_2_O_2_ in methanol for 15 min and washed in PBS 3 times for 5 min to inactivate endogenous peroxidase. Afterward, the sections were incubated with 10 μg/ml protein kinase K for 20 min at room temperature. Following washing**,** the tissue sections were incubated with TUNEL reaction solution overnight in the dark chamber at 4 ℃. After 3 times washing with PBS, the samples were incubated with DAB solution for 15 min at room temperature, and then after being washed with PBS, hematoxylin staining was done, and washing was performed subsequently. Finally, the cells with brown nuclei were evaluated as TUNEL-positive cells (20, 24). 

All the sections were examined and photographed. Photos taken were transferred to the computer, and then, using a rectangular counting grid placed randomly on the investigated photos, TUNEL-positive cells per unit area (NA) in different ovary regions were counted.

The mean numbers of TUNEL positive cells per unit area (NA) were calculated using the following formula:



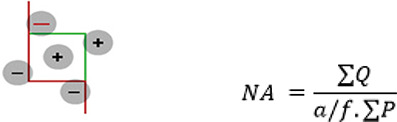



Where “NA” is the number of TUNEL positive cells per region area, the total number of counted TUNEL positive cells, a/f represents the surface area of ​​each counting frame, and ΣP is the total number of frame counts colliding with the sections (25). 

***Molecular assessment***

***RNA extraction and cDNA synthesis***

In all tissues, total RNA was extracted according to the kit manufacturer’s instructions (Pars Tous, Iran). The optical density value of RNA was measured by a Nano-drop spectrophotometer (Thermo Fisher Scientific, USA) at 260 and 280 nm. Afterward, The cDNA synthesis was performed using a cDNA synthase kit (Pars Tous, Iran) (26). The primers were designed and were ordered to Pishgam Company (Iran) (Table 1).

***Real-time PCR***

The real-time PCR was performed to evaluate the level of *CCND1* and *PCNA* gene expression by Light cycler 96 system (Roche, Switzerland), using the SYBR green master mix (Pars Tous, Iran). The *GAPDH* gene was used as internal control and a housekeeping gene. Each experiment was repeated in duplicate, and the relative fold-changes of genes were calculated by the 2^-ΔΔCt^ method (27).

***Statistical analysis***

The statistical analysis was done by the GraphPad Prism software (version 8.0; GraphPad Software). We used the One-Way ANOVA statistical test following Tukey’s *post hoc* test to evaluate the significant differences between groups. For all tests, *P<*0.05 was considered significant.

## Results

***Hormone assessment results***

***AMH level***

**Figure 2:**
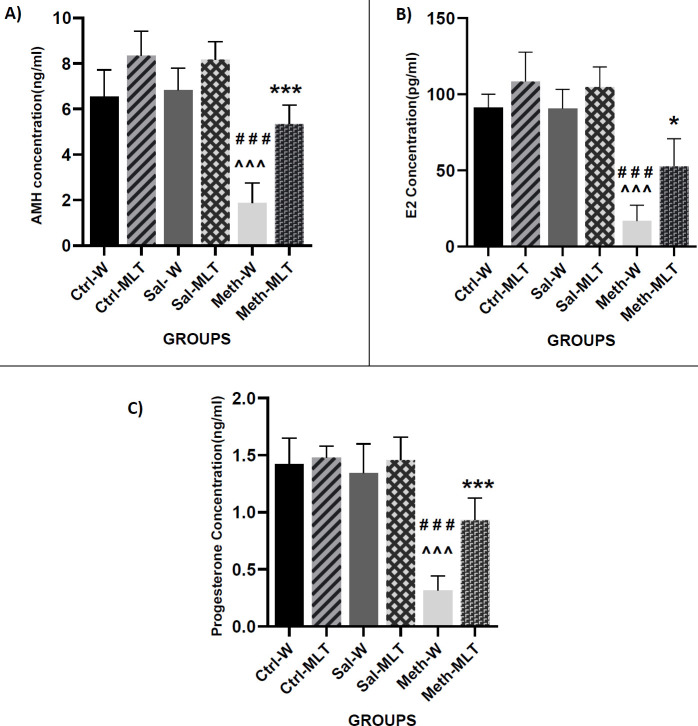
Comparison of the plasma level of AMH (A), E2 (B), and progesterone (C) in different studied groups. ### *P*<0.001 in comparison with the control groups. ^^^ *P*<0.001 in comparison with the saline groups. *** *P*<0.001 and * *P*<0.05 in comparison with the METH-W group, Mean ± SEM, n=4

AMH: Anti-Müllerian hormone; METH: Methamphetamine

Our results showed that the level of AMH in the METH-W group was decreased compared to Ctrl-W, Ctrl-MLT, Sal-W, and Sal-MLT groups (*P<*0.001), and in Ctrl-MLT and Sal-MLT groups was significantly increased compared **to** the METH-MLT group (*P<*0.001) and (*P<*0.01) respectively. The level of AMH in the METH-MLT group was higher than the METH-W group (*P<*0.001). There was no significant difference between METH-MLT, Ctrl-W, and Sal–W groups (Figure 2A).

***Estrogen level***

The estrogen level in the METH-W group **significantly** declined compared to Ctrl-W, Ctrl -MLT, Sal-W, and Sal-MLT groups (*P<*0.001), and in the METH-MLT group was significantly lower compared to Ctrl-MLT, Sal-MLT (*P<*0.001) Ctrl-W, and Sal-W groups (*P<*0.01). The estrogen level in the METH-W group was lower than the METH-MLT group (*P<*0.05) (Figure 2B).

***Progesterone level***

The obtained results showed that the level of progesterone was significantly reduced in the METH-W group compared to Ctrl-W, Ctrl-MLT, Sal-W, and Sal-MLT groups (*P<*0.001), and in the METH-MLT group was significantly lower than Ctrl-MLT, Sal-MLT (*P<*0.01), Ctrl-W, and Sal-W groups (*P<*0.05). The level of progesterone in the METH-MLT group was considerably increased in comparison to the METH-W group (*P<*0.001) (Figure 2C).

***Biochemical assessments results***

***MDA level***

**Figure 3:**
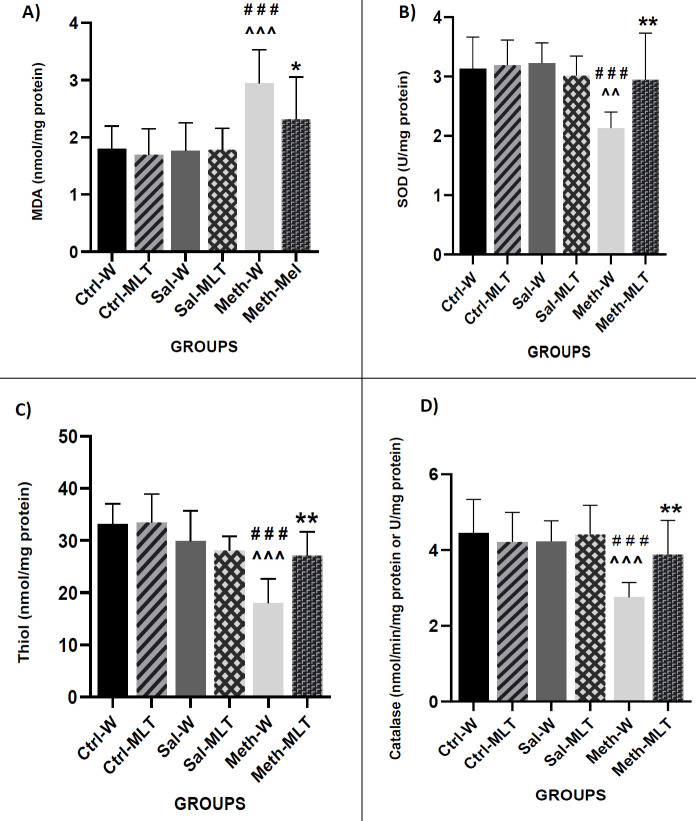
Comparing the MDA concentration (A), the activity of superoxide dismutase enzyme (SOD) (B), Thiol concentration (C), and catalase enzyme activity (D) in ovarian tissue in different studied groups. ### *P*<0.001 compared to the control groups. ^^^ *P*<0.001 and ^^ *P*<0.01 compared to saline groups. ** *P*<0.01 and * *P*<0.05 in comparison with the METH-W group, Mean ± SEM, n=4

The result of the measurement of MDA concentration in ovarian tissue showed that the MDA level in the METH-W group was significantly increased compared to Ctrl-W, Ctrl-MLT, Sal -W, and Sal-MLT groups (*P<*0.001), and in the METH-MLT group it was lower than the METH-W group (*P<*0.05) (Figure 3A).

***SOD level***

The SOD level in the METH-W group was decreased in comparison with Ctrl-W, Ctrl-MLT groups (*P<*0.001), Sal-W, and Sal-MLT groups (*P<*0.01), and in the METH-MLT group, it was higher than the METH-W group (*P<*0.01) (Figure 3B).

***Thiol level***

Evaluation of thiol concentration showed that thiol concentration in the METH-W group was significantly reduced compared to Ctrl-W, Ctrl-MLT, Sal -W, and Sal-MLT groups (*P<*0.001). Furthermore, the thiol concentration in the METH-MLT group was significantly increased compared to the METH-W group (*P<*0.01) (Figure 3C).

***CAT level***

 The results showed that CAT activity in the METH-W group was significantly lower than in Ctrl-W, Ctrl-MLT, Sal-W, and Sal-MLT groups (*P<*0.001) and in the METH-W group, was it notably decreased compared to METH-MLT group (*P<*0.01) (Figure 3D).

***Histological results***

**Figure 4:**
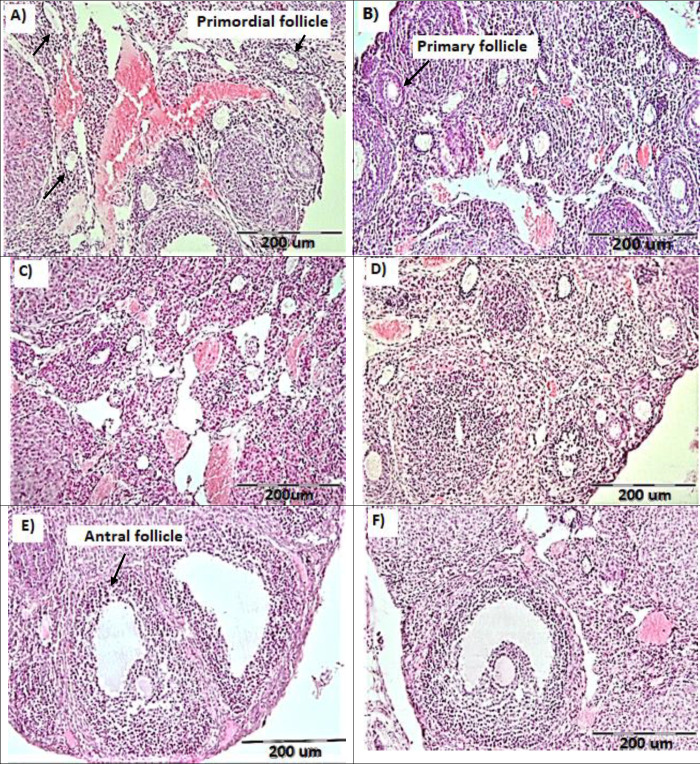
Photomicrographs of ovarian tissue sections stained with Hematoxylin and eosin (H &E) in different studied groups. A= Ctrl-W group, B= Ctrl-MLT group, C=Sal-W group, D=Sal-MLT group, E= METH-W group and F= METH-MLT group

The image of H&E of all studied groups is shown in Figure 4.

***Follicle counting results***

*Primordial follicles*

**Figure 5:**
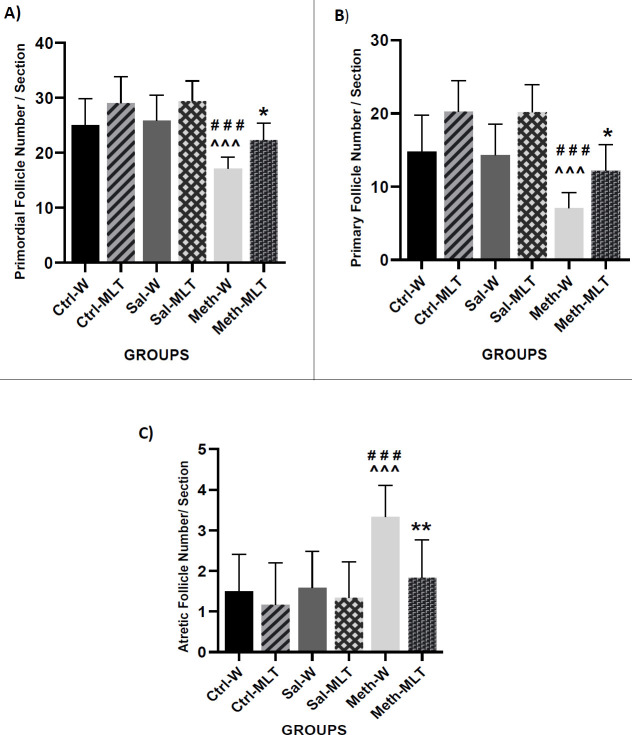
Comparing the number of the primordial follicles (A), primary follicles (B), and artistic follicles (C) in different studied groups. ### *P*<0.001 in comparison with the control groups. ^^^ *P*<0.001 in comparison with the saline groups. ** *P*<0.01 and *P<0.05 in comparison with the METH-W group, Mean ± SD, n=4

Based on Figure 5A, there was no significant difference in the number of primordial follicles between Ctrl-W, Sal-W groups, and Ctrl-MLT, Sal-MLT groups. The number of primordial follicles in the MTEH-W group was significantly decreased compared to Ctrl-W, Ctrl–MLT, Sal-W, and Sal- MLT (*P<*0.001), and in METH-MLT it was decreased in comparison with Ctrl–MLT (*P<*0.01) and Sal- MLT (*P<*0.001). The number of primordial follicles in the METH-MLT group was significantly higher than in the METH-W group (*P<*0.05, Figure 5A).

*Primary follicles*

The number of primary follicles in the Ctrl-MLT and Sal-MLT groups was significantly higher than in Ctrl–W (*P<*0.05) and Sal-W (*P<*0.01) groups. The number of primary follicles in the METH-W group was significantly less than Ctrl -W, Ctrl -MLT, Sal-W, and Sal–MLT groups (*P<*0.001). The number of primary follicles in the METH-W group was significantly decreased in comparison with the METH-MLT group (*P<*0.05). There was no significant difference between the MTEH-MLT, Ctrl -W, and Sal-W groups. Results showed a significant change in the number of primary follicles in the Ctrl-MLT and Sal- MLT groups in comparison with the METH-MLT group (*P<*0.001, Figure 5B).

*Atretic follicles*

The number of atretic follicles in the METH-W group was significantly increased in comparison with the Ctrl-W, Ctrl-MLT, Sal -W, and Sal-MLT groups (*P<*0.001) and in the METH-MLT group, it was significantly lower than the METH-W group (*P<*0.01, Figure 5C). 

***TUNEL-positive cells assessment***

**Figure 6:**
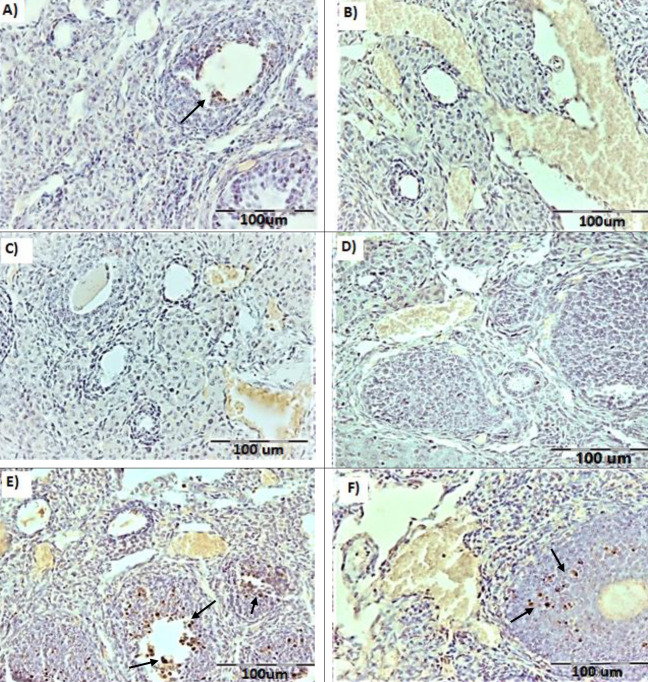
Photomicrographs of the TUNEL-positive cells of ovarian tissue sections in different studied groups. A= Ctrl-W group, B= Ctrl-MLT group, C=Sal-W group, D=Sal-MLT group, E= METH-W group, and F= METH-MLT group. Dark brown nuclei are TUNEL-positive cells

**Figure 7:**
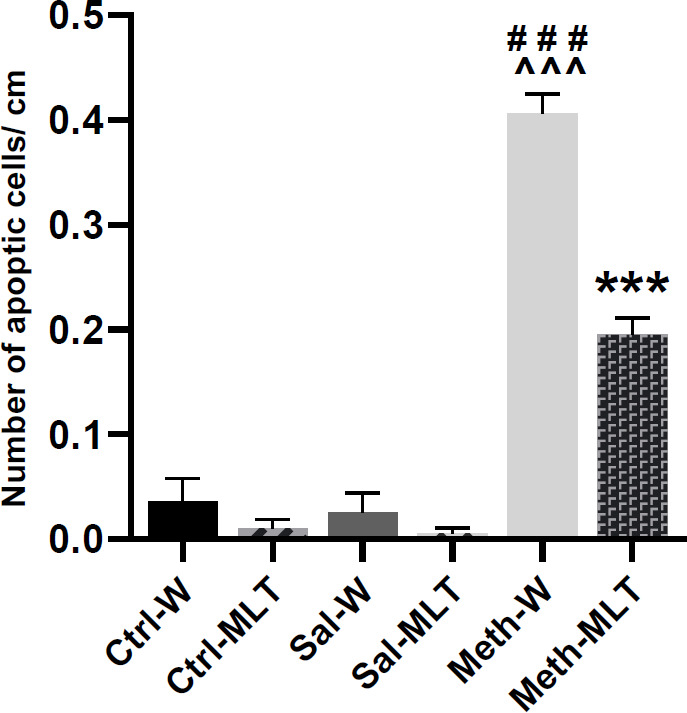
Comparison of the mean number of TUNEL-positive cells in different studied groups. ### *P*<0.001 compared to control groups. ^^^ *P*<0.001 compared to saline groups. *** *P*<0.001 compared to the METH-W group, Mean ± SEM, n=4

The TUNEL-positive cells were identified as cells with a dark brown nucleus (Figure 6). The results of the TUNEL staining showed that the mean number of TUNEL-positive cells in the METH-W and METH-MLT groups was increased compared **to** the Ctrl -W, Ctrl-MLT, Sal-W, and Sal-MLT groups (*P<*0.001). The mean number of the TUNEL-positive cells in the METH-MLT group was decreased compared **to** the METH-W group (*P<*0.001, Figure 7).

***Molecular assessment results***

*CCND-1 expression*

**Figure 8:**
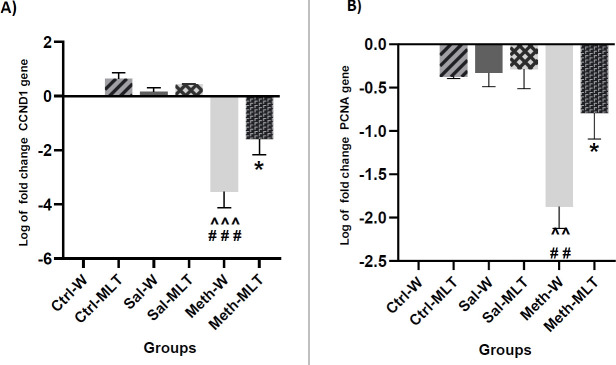
Comparing the expression of CCND1 gene (A) and PCNA gene (B) in different groups. ### *P*<0.001 and ## *P*<0.01 in comparison with the control groups. ^^^ *P*<0.001 and ^^ *P*<0.01 compared to saline groups. *P<0.05 compared to the METH-W group

METH: Methamphetamine; PCNA: proliferating cell nuclear antigen; CCND1: CCND1 Cyclin D1

The expression of the *CCND-1* gene in the METH-W group was lower than in the Ctrl-W, Ctrl-MLT, Sal-W, and Sal-MLT groups (*P<*0.001), and the Ctrl-W, Sal-W groups had higher *CCND-1* gene expression compared to the METH-MLT group (*P<*0.05). The *CCND-1* gene expression in the METH-MLT group was decreased in comparison with the Ctrl-MLT and Sal-MLT groups (*P<*0.01). The METH-MLT group had higher *CCND-1* gene expression compared to the METH-W group (*P<*0.05, Figure 8A).

*PCNA expression*

The expression of the *PCNA* gene in the METH-W group was reduced compared to the Ctrl -W, Ctrl-MLT, Sal-W, and Sal-MLT groups (*P<*0.01). The *PCNA* gene expression in the METH-MLT group was higher than in the METH-water group (*P<*0.05, Figure 8B).

## Discussion

METH, as a psychotropic and stimulant drug, increases sexual behavior in men and women (28). As a global health concern, METH abuse during pregnancy affects mothers and neonatal health. (29). Melatonin is a lipophilic hormone with anti-oxidant activity that impacts numerous metabolic processes and fertility (30). Some studies have investigated the effect of METH abuse during pregnancy and lactation on the quality of ovarian tissue in offspring (2), but based on our knowledge, there is no research about the treatment to reduce the adverse effects of METH on the reproductive organs of the next generation. The number of primordial follicles is known as an important factor for evaluating ovarian reserve (31). Despite the proliferation of germ cells, the onset of oocyte meiosis, and the formation of primordial follicles occurring during the embryonic period, only one study investigated the genes involved in ovarian developmental signaling in the embryonic period that is disrupted by parental smoking (32). 

In this study, the number of primordial follicles decreased in the METH-W group. Moreover, the primary follicles were reduced in the METH-W group, while the number of atretic follicles increased in this group. Our findings were approved by a previous study that reported METH abuse reduced the number of primordial and primary follicles and increased the number of atretic follicles (2). According to our study, the number of primordial and primary follicles was increased in the METH group treated with melatonin compared to the METH-W group. In the case of primary follicles, control and saline groups treated with melatonin were significantly increased compared to the Ctrl-W and Sal-W groups, and melatonin can effectuate the maturation of follicles and ovulation (30). In addition, in the METH-MLT group, the number of atretic follicles decreased compared to the METH-W group. A previous study indicated a gradual decrease in the quality and quantity of ovarian follicles and oocytes following ovarian aging. Hence, melatonin treatment can inhibit ovarian aging, but this mechanism isn’t completely understood. According to this previous study, melatonin as an anti-oxidant protects the ovarian quality and quantity reduction to maintain the fertilization ability. Its function may be related to telomerase, the SIRT family activity, ribosome function, and autophagy, but the molecular mechanism of the effect of melatonin on ovarian aging demands further studies (33).

AMH also demonstrates ovarian reserve, the level of AMH in plasma is related to primordial follicle numbers. Granulosa cells of growing and non-atretic follicles produce AMH. AMH inhibits the development of primordial follicles, so it decelerates the rate of ovarian depletion (34). In the current study, the level of AMH hormone in the METH-W group significantly declined, which was confirmed by Wang *et al.*, who reported significantly reduced concentrations of AMH in the METH abuser and also, METH abuse during adolescent periods had an impact on ovarian reserve in their adulthood (2). The level of AMH in the METH group treated with melatonin increased. A previous study reported that melatonin leads to an increase in the level of AMH and reduces the atretic follicles (35). METH abuse disrupts the function of the hypothalamic-pituitary-gonadal axis, so the abnormal menstrual cycle increase in METH abuser women (6). The estrogen and progesterone concentration have shown significant reduction in the METH-W group. While in the METH-MLT group, the serum estrogen and progesterone levels were significantly more than in the METH-W group. Even in males, METH administration causes a decrement in the expression of progesterone and estrogen receptors in the testis (4). 

Based on our study, in the METH-W group, the level of MDA has significantly increased, and the SOD, CAT, and thiol levels decreased, so METH abuse induces oxidative stress in ovarian tissue. It is in agreement with Xiong’s study that indicated higher levels of MDA and lower levels of SOD in the METH group (36). Some studies have demonstrated that METH induces the increasing activity of SOD in the nigrostriatal area of the brain. This enhancement in SOD activity may be a result of METH neurotoxicity response (37) and decreased CAT activity (38) because the SOD converts the O_2_^- ^to H_2_O_2,_ and the CAT converts the H_2_O_2_ to H_2_O (37). Melatonin, as an anti-oxidant, was able to reduce the oxidative stress induced by METH abuse. Similar to another study, our results prove that melatonin can increase the activity of CAT, SOD, and thiol, so it has a protective impact on oxidative stress. In addition, melatonin has an effective role in declining MDA levels, a product of lipid peroxidation of the cell membrane (30).

METH abuse leads to mitochondrial dysfunction followed by increased oxidative stress and pro-apoptotic factor (37). In higher doses of ROS, P53 induces apoptosis by down-regulating the transcription of pro-survival proteins such as Bcl-2, Bcl-XL, and IAPs, and upregulating the transcription of pro-apoptotic proteins in the intrinsic pathway such as Bax, Bid, Puma, Noxa, and Apaf-1, and extrinsic pathway including Fas, FasL, DR-4, and DR-5 (39). Based on current results, METH administration during the pregnancy and lactation period induces ovarian cell apoptosis in offspring, while melatonin decreases it. Our results are in line with Yangs *et al*. study that reported melatonin has an anti-apoptotic impression and decreases the positive TUNEL cells (40). PCNA is known as a proliferation marker in the granulosa cells. It plays an essential role as a primary regulator in developing ovarian follicles. Moreover, PCNA is effective in biological processes involving DNA replication, DNA repair and cell cycle control (41). CCND1 is related to the regulation of the cell cycle. Also it can motivate granulosa cell proliferation in the ovarian follicles (42). Our finding indicated a decrement in the expression of *PCNA* and *CCND1* genes after METH abuse, whereas in the group treated with melatonin, the expression of these genes enhanced. Consistent with our results, a previous study has demonstrated an increase in cell proliferation in rat ovaries following melatonin treatment (30). 

The results of our study showed METH administration during pregnancy, and lactation can affect ovarian reserve in the next generation. Melatonin may play a critical role in eliminating these adverse effects. Although we emphasized these effects during early maturation, these may be temporary and suppressed in adulthood, so further studies are suggested. Also, more studies with different doses of melatonin and increasing the duration of treatment can be of great interest and may impact the results.

METH: Methamphetamine

**Table 1:** Primers used for real-time PCR (5’-3’)

Product size	Genes primer sequence (5’–3’)	TM	Genes
101	GGGGTCCCAGCTTAGGTTC	59.39	m-GAPDH-F
	CCCAATACGGCCAAATCCG	58.97	m-GAPDH-R
183	GCGTACCCTGACACCAATCTC	60.74	m-CCND1-F
	CTCCTCTTCGCACTTCTGCTC	60.74	m-CCND1-R
168	AGATGTGCCCCTTGTTGTAGAG	60.03	m-PCNA-F
	GAAAAGACCTCAGGACACGC	58.85	m-PCNA-R

m: Mouse, F: Forward, R: Reverse, TM: Temperature of melting

MDA: Malondialdehyde METH: Methamphetamine

METH: Methamphetamine

METH: Methamphetamine

METH: Methamphetamine

METH: Methamphetamine

## Conclusion

It can be concluded that METH abuse during pregnancy and lactation induces oxidative stress and apoptosis in the ovarian tissue of offspring and affects the ovarian reserve by reducing primordial follicles and AMH. The administration of melatonin as an anti-oxidant agent in the next generation can alter these adverse effects of METH in the ovaries of the next generation.

## Authors’ Contributions

ON, KS, and AEB Designed the experiments; ON, SK and, BA Performed experiments and collected data; ES, OR, AEB and SK Discussed the results and strategy; AEB and ES Supervised, directed and managed the study; ES, SK, and AEB Final approved of the version to be published.

## Conflicts of Interest

The authors declare that they have no conflicts of interest.
